# Progress in Surgery

**Published:** 1897-04-24

**Authors:** 


### Progress in Surgery.

### SURGERY OF THE SPINE.

**Injuries of the Spine and Cord.**—As in so many other departments of surgery, the aid of the Röntgen rays has been recently called in to establish or confirm a diagnosis in cases of spinal injury. A case is reported by Whittaker,[1] in which a married woman, aged 22, while carelessly manipulating a loaded pistol received the discharge in the abdomen. She immediately collapsed, vomited blood, but did not become unconscious. The lower part of the body at once became paralysed, perception of touch and pain were lost below the level of the stomach, and the bladder and bowels were paralysed. Bed sores appeared on points of pressure within a few days. Five weeks after the accident she was still absolutely paraplegic, with loss of all reflexes and large deep bed sores at various points.

By means of the X-rays the bullet was localised in the body of the eighth dorsal vertebra, whence it was removed by operation. It was found that fragments of bone were pushed into the spinal canal, and pressed upon the cord. The patient recovered.

In a clinical lecture, Victor Horsley[2] recently drew attention to some points of surgical interest in regard to spinal injuries. He emphasised the danger there is of diagnosing lesions too low. As the posterior roots enter the cord they divide into an ascending channel and a descending one. It is probable that the descending channel is concerned with reflex movements, and therefore the representation of various reflexes can well be apparently lower than the entry of the nerve root. As regards the question of operation for spinal injuries, Horsley advises in acute cases that we should wait if the lesion is in the cervical region, but not if it is in the lumbar or dorsal region. The most important question is whether the injury is recent or of long standing. If recent, in the cervical region, wait; in the dorsal region, if there is not evidence of continued compression, wait a little; in the lumbar region there is no advantage in waiting. In long-standing cases surgical interference depends on whether the cord is completely divided or not. Absolute destruction of the cord has never been followed by regeneration, while with partial destruction recovery often takes place with remarkable rapidity.

Hæmorrhage into the cord also materially affects the decision. It is recognised by the presence of analgesia and muscular atrophy. Vasomotor paralysis also helps. It exhibits itself as a pink blush, with a swelling of the skin; the limb is œdematous through stagnation of the lymphatics, but does not readily pit on pressure. This symptom contra-indicates operation, because it means complete division of the cord. When there is reason to believe that there is compression exerted by fragments of bone an operation should be done at once, otherwise it is better to try extension and fixation for a time, and to operate later if necessary.

**Laminectomy for Simple Fractures.**—In a paper read before the New York Surgical Society, Gallaudet[3] reports three cases in which laminectomy was performed for simple fracture, and draws attention to some points in connection with this operation. Laminectomy is never indicated unless the regular symptoms of compression of the cord or cauda equina are present. The mortality from the operation is very much lower when the lesion is in the lower dorsal and lumbar region than elsewhere. This is especially true of cases where the operation has been done for simple fracture. The diagnosis of simple fracture in most cases is easy, especially in the lumbar region. The writer considers that the sooner the operation is done the better, once a clear diagnosis has been made, and the lesion localised. As regards the writer's own three cases, two of which were lumbar and one upper dorsal, all of them presented the regular symptoms of total loss of power and sensation in the lower extremities, areas of anæsthesia on the trunk according to the height of the lesion, incontinence of urine and fæces, and all developed bed-sores within two or three days. The diagnosis was readily made by palpation in all three, and in the lumbar cases the operation was performed within forty-eight hours of the receipt of injury. The dorsal case was fatal. The other two patients lived, but were only partially relieved by the operation. The writer emphasises two points related to the operative technique: (1) The evil effects of using the chisel and mallet in raising fractured bone; and (2) the uselessness of opening the dura unless one is certain of the presence of an intradural hæmorrhage.

**Wounds of the Cord.**—The conclusions arrived at by Enderlen[4] from an experimental study on animals are: (1) That degeneration extends to parts of the cord adjacent to the seat of injury. (2) The amount of degeneration is proportionate to the degree of injury inflicted, but its extension is not always uniform. (3) Swollen axis cylinders may be found as late as the thirty-fifth day. (4) The ganglion cells degenerate in the region of the wound, they increase above and below it. The grey matter of the cord rapidly returns to normal. (5) In no instance has regeneration taken place after injury. (6) Degeneration has followed the injection of fresh blood clot, and the introduction of small particles of kidney below the dura. This he attributes not to pressure, but to disturbance of the circulation in the cord.

Wagner[5] considers the essential lesion in spinal concussion is in the structure of the nerve cells, and that this alteration is followed by secondary degenerations.

[1] Internat. Med. Mag., Nov., 1896. [2] Clin. Jour., Jan., 1897. [3] Therap. Gaz., Nov., 1896. [4] Deutsche Zeitschrift f. Chirurg., 1896. [5] Beiträge zur Klin. Chirurgie., Ba. XII.

